# PLAC8 promotes adriamycin resistance via blocking autophagy in breast cancer

**DOI:** 10.1111/jcmm.16706

**Published:** 2021-06-11

**Authors:** Yongxia Chen, Yunlu Jia, Misha Mao, Yifeng Gu, Chenpu Xu, Jingjing Yang, Wenxian Hu, Jun Shen, Dengdi Hu, Cong Chen, Zhaoqing Li, Lini Chen, Jian Ruan, Peng Shen, Jichun Zhou, Qun Wei, Linbo Wang

**Affiliations:** ^1^ Department of Surgical Oncology Sir Run Run Shaw Hospital Zhejiang University School of Medicine Hangzhou China; ^2^ Department of Medical oncology the First Affiliated Hospital Zhejiang University School of Medicine Hangzhou China; ^3^ Department of Internal Medicine UT Southwestern Medical Center Dallas TX USA

**Keywords:** adriamycin resistance, autophagy, breast cancer, p62, PLAC8

## Abstract

Adriamycin (ADM) is currently one of the most effective chemotherapeutic agents in breast cancer treatment. However, growing resistance to ADM could lead to treatment failure and poor outcome. PLAC8 was reported as a novel highly conserved protein and functioned as an oncogene or tumour suppressor in various tumours. Here, we found higher PLAC8 expression was correlated with worse outcome and aggressive phenotype in breast cancer. Breast cancer patients with higher PLAC8 expression showed potential ADM resistance. In vitro experiments further confirmed that PLAC8 inhibited by siRNA or enforced overexpression by infecting pcDNA3.1(C)‐PLAC8 plasmid correspondingly decreased or increased ADM resistance. Subsequently, we demonstrated that ectopic PLAC8 expression in MCF‐7/ADMR cell blocked the accumulation of the autophagy‐associated protein LC3 and resulted in cellular accumulation of p62. Rapamycin‐triggered autophagy significantly increased cell response to ADM, while the autophagy inhibitor 3‐MA enhanced ADM resistance. 3‐MA and PLAC8 could synergistically cause ADM resistance via blocking the autophagy process. Additionally, the down‐regulation of p62 by siRNA attenuated the activation of autophagy and PLAC8 expression in breast cancer cells. Thus, our findings suggest that PLAC8, through the participation of p62, inhibits autophagy and consequently results in ADM resistance in breast cancer. PLAC8/p62 pathway may act as novel therapeutic targets in breast cancer treatment and has potential clinical application in overcoming ADM resistance.

## 1 INTRODUCTION

Breast cancer is the leading cause of cancer‐related death in women aged from 20 to 59 years.^1^, ^2^ Adriamycin (ADM), also known as doxorubicin, is a DNA topoisomerase II inhibitor and belongs to the family of anthracycline anticancer drugs. ADM is used as one of the most effective agents in breast cancer clinical treatment.^3^ Unfortunately, chemotherapeutic resistance to ADM has emerged as one of the essential reasons for treatment failure and death associated with breast cancer.^4^, ^5^ Significant efforts should be undertaken to resolve the molecular mechanisms that lead to ADM resistance and identified novel biomarkers that can predict treatment response and overcome ADM resistance.

Placenta‐specific 8 (PLAC8, Onzin) was firstly identified via genome‐wide gene expression analysis in the placenta, and PLAC8 protein was restricted to the spongiotrophoblast layer of the mouse placenta.^6^, ^7^ Multiple functions of PLAC8 have been demonstrated in the regulation of immunity, adipocyte differentiation and human cancers.^8^, ^9^, ^10^, ^11^, ^12^, ^13^ Previously research revealed that PLAC8 is involved in the progression of various cancers. For example, PLAC8 recovery inhibited PI3K/Akt/GSK3b/Wnt/β‐catenin signalling to reduce cell proliferation in liver carcinoma.^8^ The overexpression of PLAC8 was also revealed to be associated with malignant progression and poor prognosis in clear cell renal cell carcinoma.^9^ In colon cancer, PLAC8‐overexpressing cells exhibited increased phosphorylated ERK2, which elevated cell motility and cancer invasion.^10^ From our two previously published studies, PLAC8 was relatively highly expressed in lung cancer and breast cancer tissues, and contributed to cancer progression.^11^, ^12^ Besides, several studies have demonstrated the role of PLAC8 in the regulation of autophagy during pancreatic cancer progression and prostate carcinogenesis.^13^, ^14^

Recent studies have revealed an association between autophagy and drug resistance. Autophagy is a homeostatic, catabolic degradation process, by which the damaged or unwanted organelles and protein aggregates are delivered to lysosome for degradation.^15^ Autophagy is essential for sustain proper function and metabolism of the cell. The involvement of autophagy in ADM resistance has been extensively investigated. Some studies indicated that autophagy has a protective role in cancer cells and results in ADM resistance. Other observation, however, showed that induction of autophagy helps overcome ADM resistance and leads to cancer cell death. In breast cancer MDA‐MB‐231 cells, treatment with ADM induced cytoprotective autophagy, and Atg5‐deficient caused decreased sensitivity towards ADM.^16^

The role of PLAC8 in the regulation of autophagy and ADM resistance in breast cancer has not been reported until now. In this study, we firstly reported the potential correlation between elevated PLAC8 expression level and ADM resistance in breast cancer tissues. Furthermore, enforced overexpression or inhibition of PLAC8 could modulate autophagy through p62 participation and thus altered ADM sensitivity in breast cancer cells. An in‐depth understanding of the mechanisms of PLAC8/autophagy regulation in relation to ADM resistance will provide a promising therapeutic strategy for overcoming ADM resistance of breast cancer treatment.

## 2 MATERIALS AND METHODS

### 2.1 Cell culture

Human breast cancer cell lines MCF‐7 and MDA‐MB‐231 were obtained from the Cell Bank of the Chinese Academy of Sciences. The ADM‐resistant MCF‐7 cells (MCF‐7/ADMR), which were derived from the human breast cancer cell MCF‐7, were maintained in the presence of 1 μg/ml ADM. MCF‐7 cells were treated with ADM at the starting concentration of 1 ng/ml. A double concentration of ADM was then applied when the cells became resistant to ADM. The process was repeatedly performed to increase cell tolerance to ADM. Cells were cultured in RPMI 1640 medium (Invitrogen) supplemented with 10% foetal bovine serum (GIBREAST CANCERO) and grew at 37°C with 5% CO2 and 95% humidity.

### 2.2 Sample collection and patient characteristics

Breast tissues were collected from the Department of Surgical Oncology, Sir Run Shaw Hospital, Zhejiang University. Patients with breast cancer undergo tumour tissue and normal tissue sample collection at the time of medically indicated surgery or biopsy. Clinically, ADM resistance can be observed as clinical progression or cancer recurrence during or after completion of adjuvant therapy, following surgery or in rare cases after complete pathological response following a period of drug therapy.

### 2.3 Plasmids, siRNAs, and transfection

Lentiviral PLAC8‐overexpressing and negative control vectors were obtained from GENE. Short interfering RNAs targeting PLAC8 (siPLAC8), P62 (siP62) and a scrambled control siRNA were designed and commercially synthesized by Ribo Bio. Cells were seeded 24 hours in 12‐well plates (1 × 10^5^/well) and transfected with siRNA duplexes (50 nmol/L) or 1 to 2 μg of plasmids using Lipofectamine 3000 (Invitrogen) following the manufacturer's instructions. Cells were harvested for RNA and protein extraction 48 hours after transfection and processed for functional assays.

### 2.4 RNA isolation and real‐time fluorescent quantitative PCR

Total RNA was extracted after 48 hours transfection using TRIzol reagent (Invitrogen) following the manufacturer's instruction. The concentrations were quantified by NanoDrop 2000 (Thermo Scientific, USA), and the RNA (1 μg) was reverse‐transcribed by the HiFiScript cDNA Synthesis Kit (CWBIO, CW2569M). Real‐time fluorescent quantitative PCR was performed using UltraSYBR Mixture (CWBIO, CW0957H). Glyceraldehyde 3‐phosphate dehydrogenase (GAPDH) was used as the reference gene. The following primers were used: PLAC8: 5'‐GGAACAAGCGTCGCAATGAG‐3' (sense) and 5'‐ AAAGTACGCATGGCTCTCCTT‐3' (anti‐sense); GAPDH: 5'‐TGCACCACCAACTGCTTAG‐3' (sense) and 5'‐AGTAGAGGCAGGGATGATGTTC‐3' (anti‐sense). SQSTM1(P62): 5'‐ATCGGAGGATCCGAGTGT‐3' (sense) and 5'‐TGGCTGTGAGCTGCTCTT‐3' (anti‐sense), The results were calculated using 2^‐△△Ct method.

### 2.5 Protein extraction and Western blot analysis

Total protein was extracted after 72 hours transfection using RIPA reagent (Beyotime Biotechnology, China) following the manufacturer's instruction. The following antibodies were used: PLAC8 (1:1000, Cell Signaling Technology, #13885), Ki67 (1:500, Sino Biological, 100130‐T32‐50), GAPDH (1:500, Santa Cruz, sc‐47724), actin‐HRP conjugated (1:500, Santa Cruz, sc‐47778), LC3 antibody (1:1000, SIGMA, L7543) and SQSTM1 (1:1000, Medical & Biological Laboratories, PM045) antibodies. The signals were detected with an ECL Kit (Bio‐Rad, ClarityTM Western ECL Substrate, 500 ml #1705061).

### 2.6 Co‐immunoprecipitation

Co‐immunoprecipitation (Co‐IP) was performed using protein lysates of cells transfected with siP62 and PLAC8 plasmid and antibody following standard protocol. Normal rabbit IgG served as negative control. The immunoprecipitates were immunoblotted with anti‐PLAC8 and anti‐P62 antibodies that were described previously. The immunocomplexes on the Western blot were detected by chemiluminescence and photographic films and quantified with multi‐Gauge soft (Fujifilm).

### 2.7 Immunofluorescence staining

Cells at a density of 1 × 10^5^were briefly plated in 6‐well plates, and three glass coverslips were placed onto the wells. The plates were incubated for 24‐48 hours until 30%‐40% confluence was reached. The cells were then fixed with 4% paraformaldehyde for 10 minutes at room temperature and permeabilized with 0.1% Triton X‐100. The slides were then washed three times with phosphate‐buffered saline (PBS) and blocked with 5% bovine serum albumin in PBS for 30 minutes at room temperature. The sections were incubated with a primary antibody against PLAC8 overnight. After rinsing three times with PBST for 5 minutes, and Alexa 568‐conjugated (red) goat anti‐rabbit antibody was applied (1:200 dilution; Life Technologies) for one hour at room temperature. Cell nuclei were counterstained with DAPI (4',6‐diamidino‐2‐phenylindole), and images were acquired using a Nikon laser scanning confocal microscope (Nikon Instruments Inc, Melville).

### 2.8 Cell proliferation assay

Cell proliferation assay was performed with MTT (5 mg/ml, Promega). Approximately 48 hours after transfection, the transfected cells were transferred into a 96‐well plate for one day. Cells were then treated with serial dilutions of ADM for 48 hours ours. The absorbance at 490 nm was measured using a multi‐mode reader (LD942). The IC50 (50% inhibitory concentration) value, which represents the drug concentration with 50% cell growth inhibition, was calculated by normal probability transforms based on the relationship of drug concentration and inhibition rat. Samples were prepared in triplicates, and the cell viability was determined as the mean ± SD.

### 2.9 Analysis of autophagy by flow cytometry

Cells added 5 ug/ml ADM and 3‐MA or RAPA incubated for 48 hours. The cells were washed, and the autophagic vacuoles were quantified using a cyto‐ID autophagy detection kit (Enzo, ENZ‐51031) according to the manufacturer's instructions. The signals of labelled autophagic vacuoles were analysed using a flow cytometer with an FL1 (488 nm excitation, green) channel.

### 2.10 Colony formation assays

Breast cancer cells were seeded in six‐well plates at 200 cells/well. The indicated concentrations of ADM were added the next day, and cells were left for seven days to form colonies. Colonies were stained with 0.25% crystal violet and 25% methanol in phosphate‐buffered saline solution for visualization. Colonies were counted manually and digitally using the ImageJ software (National Institute of Mental Health, Bethesda.

### 2.11 Immunohistochemical analysis

All formalin‐fixed and paraffin‐embedded tumour sections were treated with xylene and ethanol for deparaffinization and rehydration. After blocking endogenous peroxidase with 3% H2O2 in methanol, antigen retrieval was conducted by boiling the sections in sodium citrate buffer (0.1 mmol/L, pH 6.0) for 5 minutes. The sections were then blocked with 3% BSA for 30 minutes and incubated with antibodies against PLAC8 (1:200, Cell Signaling Technology, #13885), Ki67(1:100, Sino Biological, 100130‐T32‐50), P62 (1:200, Medical & Biological Laboratories, PM045) and LC3 (1:500, SIGMA, L7543) by using the GT Vision III IHC Assay Kit (HRP/DAB, rabbit/mouse‐general, two‐step, GK500710, Gene Tech, Shanghai, China) following the manufacturer's protocol. Images were acquired by polarized light microscopy (Nikon, Eclipse 80i).

### 2.12 Tumour xenograft assay

MCF‐7/ADMR cells were transfected with PLAC8 siRNA or negative control siRNA. An equal number (2 × 10^6^) of cells were resuspended in 60 μl of PBS with 40 μl of growth factor‐reduced basement membrane matrix (#356231 Corning) and then injected into four‐week‐old nude mice (n = 5). The mice were observed three times a week, and the tumour volumes were measured twice per week. Tumour volume was calculated with the formula V = 0.5 ab^2^ (*a*, longest tumour axis; *b*, shortest tumour axis). All mice were sacrificed four weeks after tumour cell injection. Tumour tissues were collected and processed for further analyses, including the immunohistochemistry analysis of PLAC8, Ki67, p62 and LC3 expression. Animal studies were reviewed and approved by the Ethics Committee for Animal Studies of Zhejiang University.

### 2.13 Statistical analysis

GraphPad Prism 6.0 software was used for statistical analysis. Data were independently collected from at least three experiments. The survival curve was conducted using the Kaplan‐Meier method with a log‐rank test. Two‐tailed unpaired Student's *t* tests were used to compare mean data. The results are presented as the mean ± SD, and *P* < .05 was considered statistically significant.

## 3 RESULTS

### 3.1 High PLAC8 expression was correlated with ADM resistance and predicted poor outcomes in breast cancer

**FIGURE 1:**
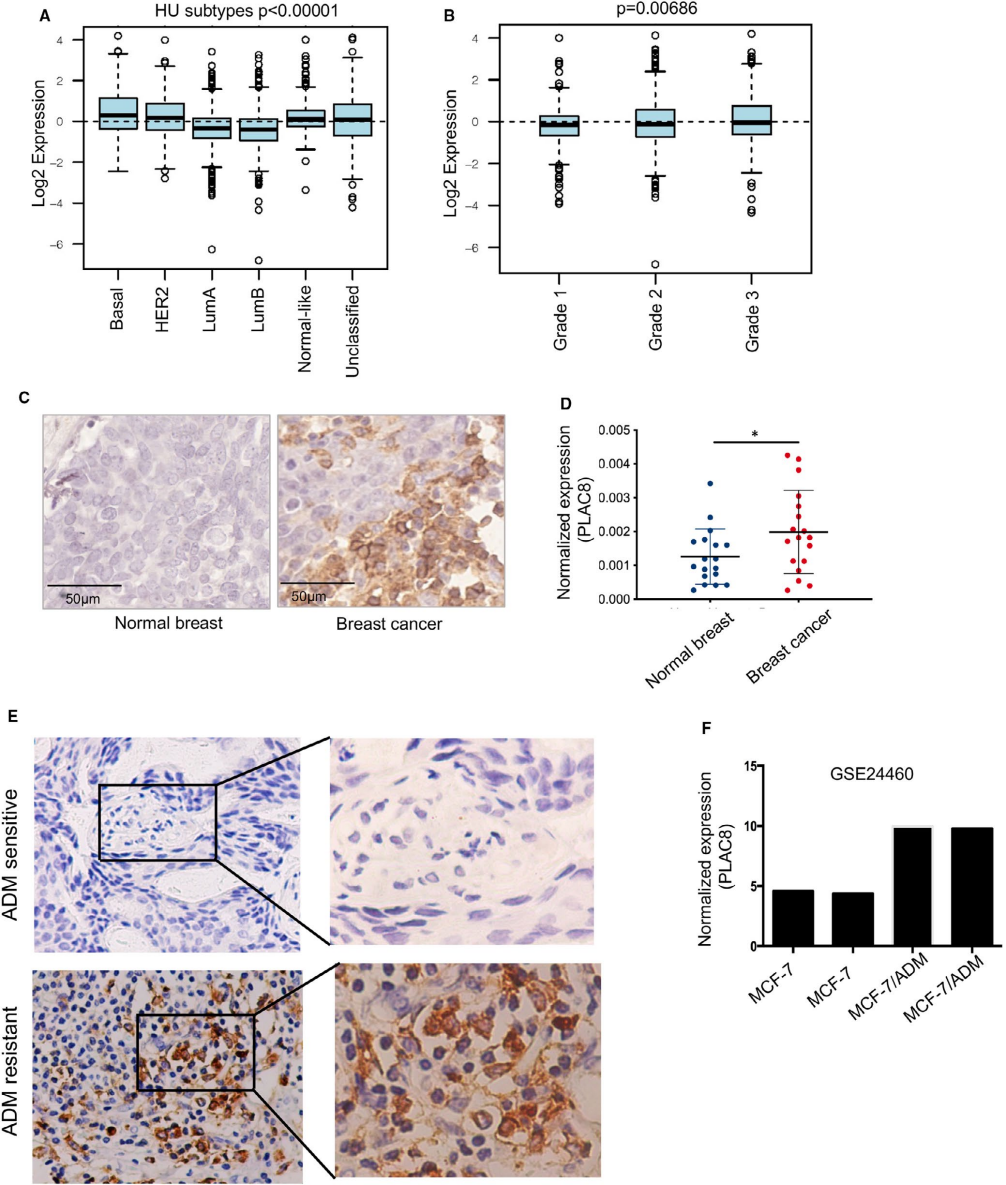
PLAC8 expression was associated with breast cancer outcome and ADM response. A, B. The correlation between PLAC8 expression levels with breast cancer subtype (B) and histological grade (C); C. Immunohistochemistry analysis of PLAC8 expression in normal breast and correspondent breast cancer tissues. Scale bar  =  50 µm; D. mRNA expression of PLAC8 between normal breast and breast cancer; E. Shown are representative images of PLAC8 staining using immunohistochemical staining for ADM resistance and ADM‐sensitive breast cancer, and PLAC8 expression was relatively increased in ADM resistance breast cancer case. F. The results from one independent GEO dataset (GSE24460) showed that PLAC8 expression was significantly up‐regulated in ADM‐resistant MCF‐7/ADMR cells compared with parental MCF‐7. Each bar represents the mean ± SD of three independent experiments. *P < .05, **P < .01, ***P < .001

Depending on the clinical tumour subtype, the triple‐negative and HER2‐positive breast cancer presented relatively higher PLAC8 expression, compared to the luminal‐type, normal‐like and unclassified type of breast cancer (*P* < .00001) (Figure 1A). Besides, the expression of PLAC8 was significantly elevated in higher histologic grade (grade2/3 compared to grade 1) (data downloaded from http://co.bmc.lu.se/gobo/gsa.pl) (Figure 1B). Compared to that in breast normal tissues, PLAC8 expression was elevated in breast cancer (Figure 1C,D).

Next, we collected clinical breast cancer specimens and divided them into ADM‐sensitive or ADM‐resistant groups. Immunohistochemistry analysis of PLAC8 protein further confirmed the previous results that ADM‐resistant breast cancer tended to be PLAC8‐overexpressed (Figure 1E). The results from the breast cancer dataset (GSE24460) also showed that PLAC8 expression was significantly up‐regulated in the resistant MCF‐7 cells compared with the parental MCF‐7 cells (Figure 1F). Here we found that PLAC8 could be a new prognostic marker in breast cancer, and there is a potential relationship between PLAC8 expression and ADM resistance.

### 3.2 A potential association between intrinsically and acquired ADM resistance and PLAC8 expression levels

**FIGURE 2:**
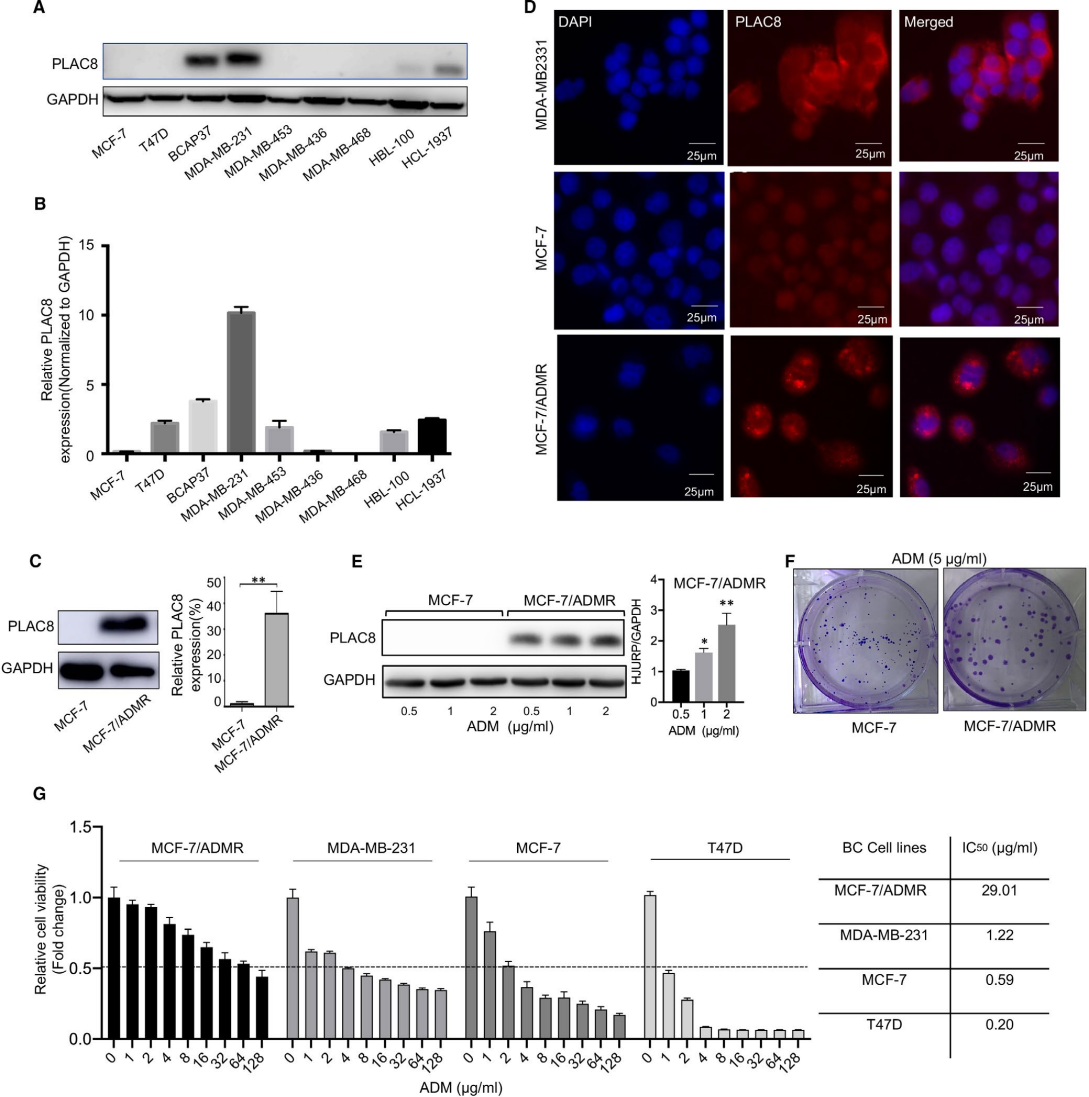
PLAC8 expression was increased in ADM resistance breast cancer cells. A, B. The protein and mRNA expression level of PLAC8 in various breast cancer cell lines. C Protein and mRNA expression of PLAC8 in MCF‐7 and MCF‐7/ADMR cells. D. Immunofluorescence staining for PLAC8 (red) and DAPI nuclear staining (blue) in MDA‐MB231, MCF‐7 and MCF‐7/ADMR cell lines. Scale bar  =  25 µm. E. Altered PLAC8 expression in MCF‐7 and MCF‐7/ADMR cell upon ADM treatment (0.5, 1 and 2 µg/ml, 48 h). F. Colony formation indicated the proliferative potential of MCF‐7 and MCF‐7/ADMR cells with ADM treatment (5 µg/ml). G. The sensitivity of MCF‐7, MDA‐MB231, T47D and MCF‐7/ADMR cells to ADM. Each bar represents the mean ± SD of three independent experiments. *P < .05, **P < .01, ***P < .001

To examine roles of PLAC8 in ADM sensitivity in breast cancer, we next tested the PLAC8 expression profile in a range of breast cancer cell lines. PLAC8 protein and mRNA expression were found to be relatively higher in MDA‐MB‐231 and BCAP37 cells (Figure 2A,2B, respectively). The cell line MCF‐7 with acquired ADM resistance and its parental MCF‐7 were used as our model system. MCF‐7/ADMR cells were established by culturing MCF‐7 cells in medium with increasing ADM till to 1 µg/ml for 6 months. Interestingly, we observed the abnormally elevated PLAC8 expression in MCF‐7/ADMR, when compared with the parental MCF‐7 cells (Figure 2C). The localization of PLAC8 in breast cancer cells (MDA‐MB231, MCF‐7 and MCF‐7/ADMR) was detected using immunofluorescence staining, and PLAC8 was localized in both the nucleus and cytosol of breast cancer cells (Figure 2D).

We next tested these breast cancer cells response to ADM, and explore the potential correlation between PLAC8 expression and ADM resistance. MCF‐7 and MCF‐7/ADMR cells were exposed to ADM at different concentrations (0.5, 1 and 2 µg/ml) for 48 hourS. The amount of PLAC8 protein was found to be substantially increased after 48 hourS exposures to ADM at various concentrations (1 and 2 µg/ml) in MCF‐7/ADMR, except for the conditions in which MCF‐7 cells treated with various concentrations of ADM (Figure 2E). As expected, colony formation assays indicated that ADM treatment led to decreased colony formation ability and smaller size of clones in MCF‐7 (Figure 2F). Furthermore, cell viability assay confirmed that MCF‐7/ADMR cells could tolerate much higher concentrations (IC50 = 29.01 µg/ml) of ADM than the parental MCF‐7 cells (IC50 = 0.59 µg/ml). Besides, the IC50 of ADM was much lower in T47D and MCF‐7 cells than MDA‐MB231 and MCF‐7/ADMR cells, suggesting that increased PLAC8 expression might play an essential role in both intrinsically and acquired ADM resistance in breast cancer cells (Figure 2G). Thus, our findings suggested that ADM induced up‐regulation of PLAC8, and the intrinsically and acquired resistance to ADM in breast cancer may partially be caused by PLAC8 overexpression.

### 3.3 PLAC8 silencing reversed breast cancer cell response to ADM

**FIGURE 3:**
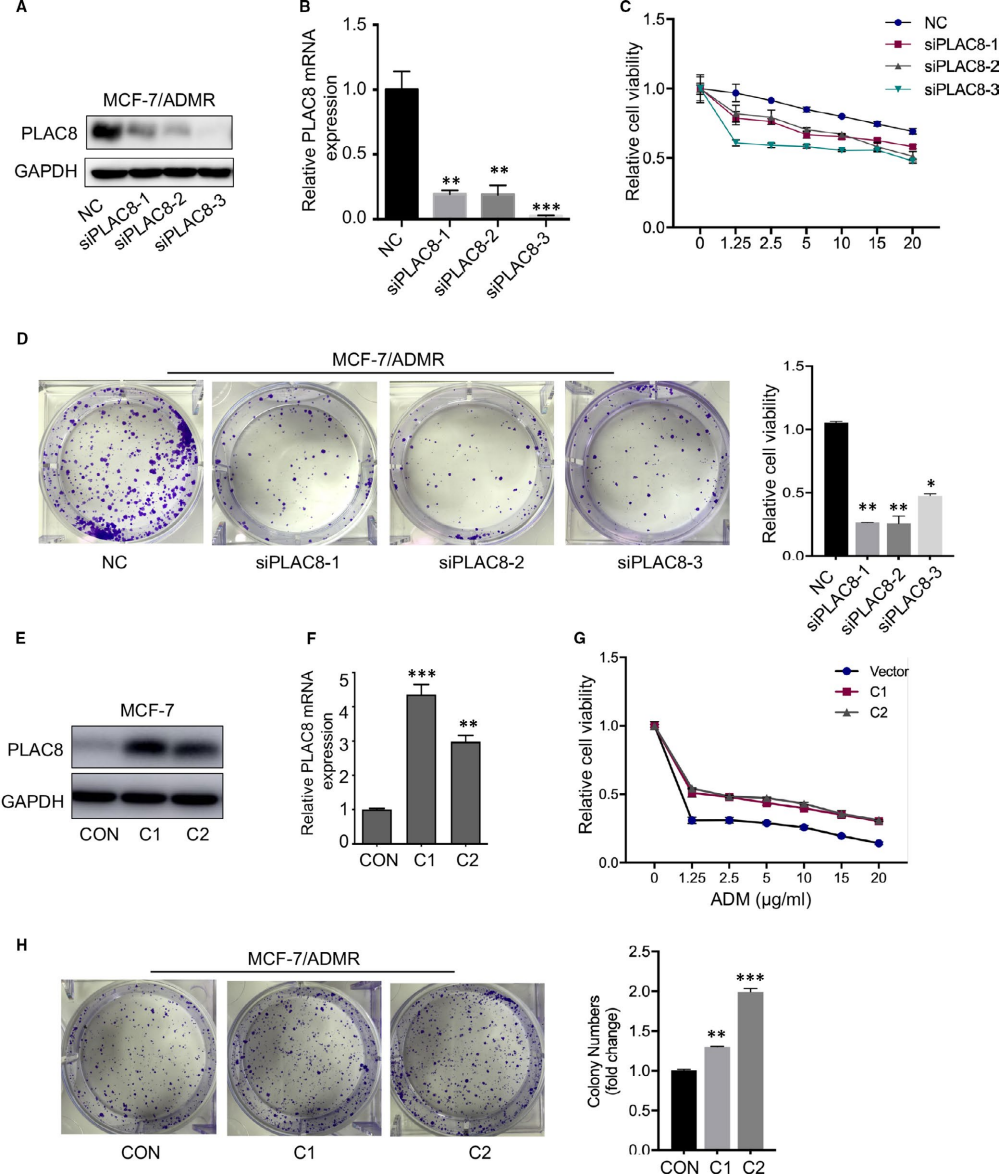
Exogenous overexpression and knockdown of PLAC8 influenced ADM sensitivity in breast cancer cells. A, B. Protein and mRNA expression of PLAC8 in MCF‐7/ADMR transfected with the control siRNA (NC), PLAC8 siRNA (siPLAC8‐1, siPLAC8‐2, siPLAC8‐3). GAPDH was used as a loading control. C. Drug sensitivity test for ADM in MCF‐7/ADMR cells infected with NC or PLAC8 siRNA (siPLAC8‐1, siPLAC8‐2, siPLAC8‐3). Cells were treated with various indicated concentrations of ADM for 48 h; cell viability upon drug treatment was analysed by an MTS assay. D. Colony formation assay were conducted to test cell response to ADM in MCF‐7/ADMR PLAC8 silencing cells, compared to the control group. E, F. Protein and mRNA expression of PLAC8 in MCF‐7 cells transfected with PLAC8 overexpression vector, and C1 and C2 were constructed‐PLAC8 stable expression cell clones. GAPDH was used as a loading control. G. Colony formation assay were conducted to test cell response to ADM in MCF‐7 PLAC8‐overexpressed cells (C1 and C2), compared to the control group. H. Drug sensitivity test for ADM in MCF‐7 cells infected with con vector or PLAC8 overexpression vector (PLAC8 OE). Cells were treated with various indicated concentrations of ADM for 48 h, cell viability upon drug treatment was analysed by an MTS assay. Each bar represents the mean ± SD of three independent experiments. *P < .05, **P < .01, ***P < .001

We further examined the effects of altered PLAC8 expression on ADM resistance in MCF‐7 and MCF‐7/ADMR cells. The knockdown experiment was performed in MCF‐7/ADMR by transfecting with PLAC8 siRNA (siPLAC8‐1, siPLAC8‐2 and siPLAC8‐3) (Figure 3A,B), and then, we tested the cell response to ADM. The sensitivity changes were explored by using MTT and colony formation assays. MCF‐7/ADMR PLAC8 silencing group become more sensitive to ADM than the control group (Figure 3C,D). Both the cell growth and colony formation ability were significantly suppressed after PLAC8 expression was inhibited. And reversely, PLAC8 overexpression by transfection with a pcDNA3.1(C)‐PLAC8 plasmid conferred protection from ADM for breast cancer cell. We overexpressed PLAC8 in MCF‐7 cell and construct the stable PLAC8 overexpression breast cancer cell (C1 and C2 clones) (Figure 3E,F, respectively) and then analysed the cell response to ADM. The results revealed that PLAC8 overexpression in MCF‐7 conferred protection from ADM for breast cancer cell MCF‐7 (Figure 3G,H). Here, we found PLAC8 silencing in MCF‐7/ADMR may be an effective way to reverse ADM resistance.

### 3.4 Autophagy activators reverse ADM resistance of MCF‐7/ADMR cell

**FIGURE 4:**
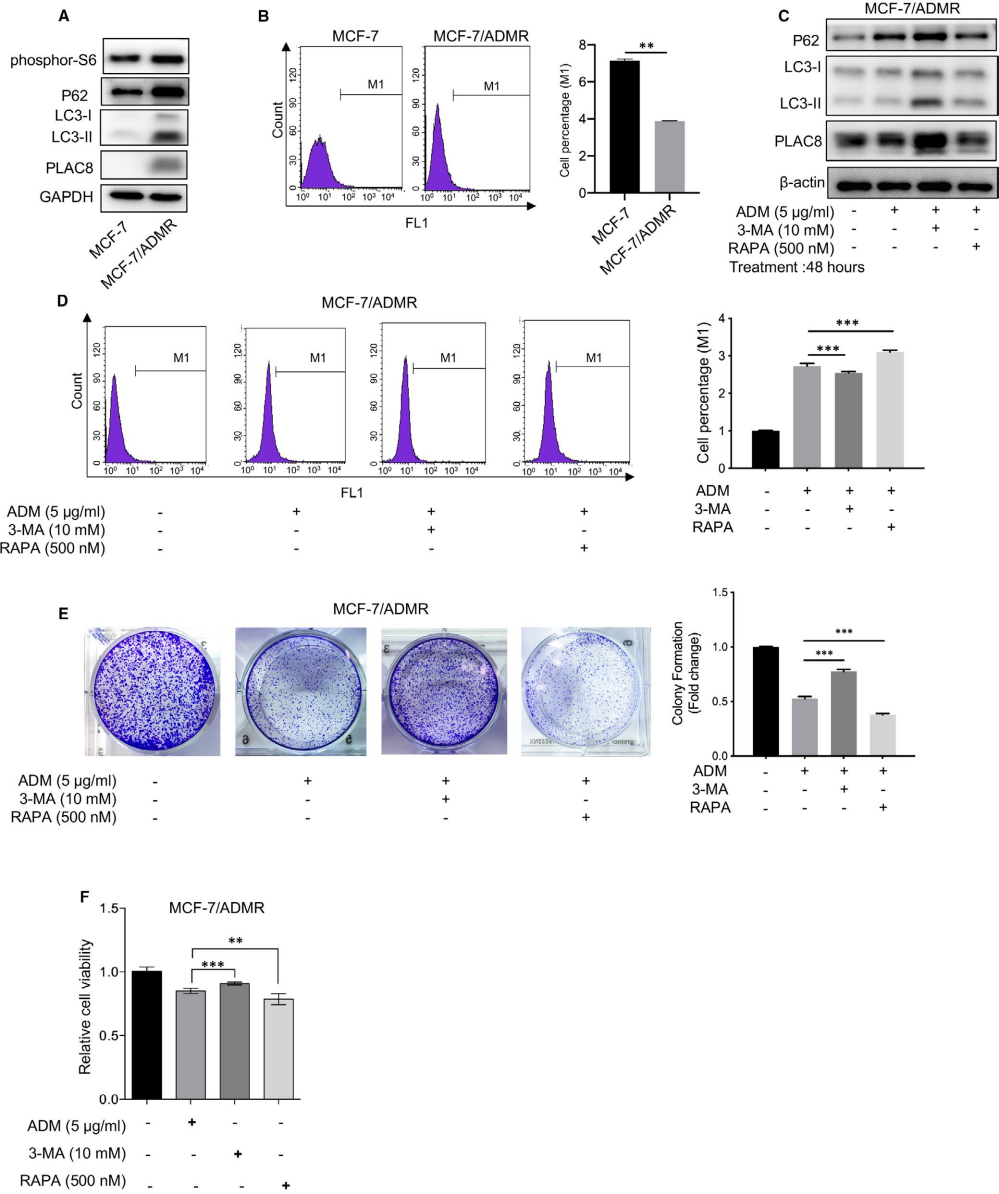
Autophagy activator re‐sensitized MCF‐7/ADMR to ADM. A. Representative immunoblots of p62, phosphor‐S6, LC3 and PLAC8 protein in MCF‐7/ADMR and MCF‐7 cells. GAPDH shown as a loading control. B. Autophagic vacuoles in MCF‐7 or MCF‐7/ADMR cells were analysed using a Cyto‐ID autophagy detection assay. The data are presented as the mean ± SD of three independent experiments. Student's *t* test was used for statistical analysis. C. Western blotting was performed to examine the expression of P62, LC3 and PLAC8 in MCF‐7/ADMR cells treated with ADM, or combined with 3‐MA or rapamycin (RAPA). **D**. Autophagic vacuoles in MCF‐7/ADMR cells were analysed using a Cyto‐ID autophagy detection assay upon treatment with ADM or combined with 3‐MA or RAPA. The data are presented as the mean ± SD of three independent experiments. Student's *t* test was used for statistical analysis. E. Colony formation assay was performed to test MCF‐7/ADMR cell response to ADM upon co‐incubation with 3‐MA or RAPA. F. Drug sensitivity test for ADM in MCF‐7/ADMR cells upon treatment with 3‐MA or RAPA. Cell viability upon drug treatment was analysed by an MTS assay. *P < .05, **P < .01, ***P < .001

There is mounting preclinical evidence that targeting autophagy can enhance the efficacy of many cancer therapies. Here, we found the autophagy flux was blocked in MCF‐7/ADMR, compared to its parental cell line. Increased LC3‐II, phosphor‐S6 and p62 accumulation were observed in the conditions of the complete medium in MCF‐7/ADMR cell (Figure 4A). The use of flow cytometry to study the autophagic process revealed that decreased autophagic cell death of MCF‐7/ADMR (Figure 4B). In addition, 3‐MA, an autophagy inhibitor, was used to inhibit autophagy in the MCF‐7/ADMR groups. The mTOR inhibitor rapamycin (RAPA) have been reported to promote the autophagy process. Here, Western blot analysis revealed increased p62 and PLAC8 protein accumulation in the 3‐MA group. In contrast, the autophagic flux in MCF‐7/ADMR was restored by RAPA preconditioning (Figure 4C,D). Thus, 3‐MA and RAPA are effective autophagy inhibitor and inducer of MCF‐7/ADMR, respectively.

Importantly, pretreatment with RAPA reversed ADM resistance and sensitized MCF‐7/ADMR to ADM therapy. However, the autophagy inhibitor 3‐MA significantly reversed the effects exerted by RAPA. 3‐MA enhanced the protective role in the ADM+ 3‐MA group, as evidenced by increased colony formation and cell growth viability (Figure 4E,F). Conclusively, our findings demonstrated that inhibition of autophagy could be exploited as a novel strategy to re‐sensitize the breast cancer cells to ADM chemotherapy.

### 3.5 PLAC8 modulates ADM resistance and autophagy through p62 participation

**FIGURE 5:**
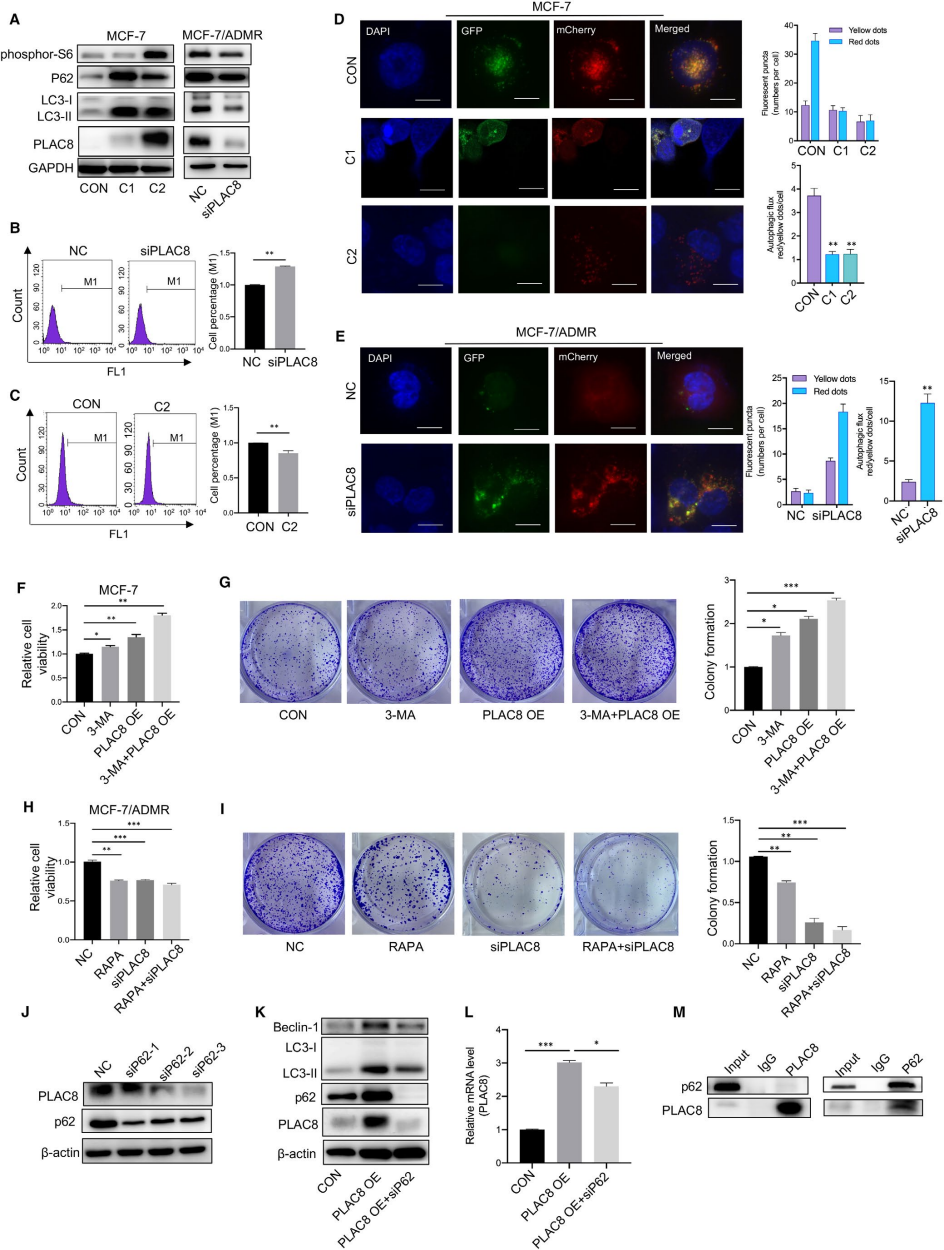
Enhanced autophagy contributed to ADM sensitivity. A. p62, phosphor‐S6, LC3 and PLAC8 expression in MCF‐7 cells that were stably infected with the PLAC8 lentiviral vector or the control lentiviral vector was analysed by Western blotting (left). p62, phosphor‐S6, LC3 and PLAC8 expression in MCF‐7/ADMR cells that were stably infected with the PLAC8 lentiviral vector or the control lentiviral vector was analysed by Western blotting (right). B. Autophagic vacuoles in MCF‐7/ADMR cells infected with the PLAC8 siRNA or the control siRNA were analysed using a Cyto‐ID autophagy detection assay. C. Autophagic vacuoles in MCF‐7 cells stably infected with the PLAC8 lentiviral vector (C2) or the control lentiviral vector were analysed using a Cyto‐ID autophagy detection assay. D, E. The distribution of autophagic vacuoles that contained mCherry‐EGFP‐LC3 in MCF‐7 cells overexpressing PLAC8 and MCF‐7/ADMR cells PLAC8 silencing was analysed by confocal microscopy. Quantification of representative pictures showing the functionality of the sensor. Manual counting of red and yellow dots was performed. Autophagic flux is estimated as the ratio between red and yellow dots. F. Drug sensitivity test for ADM in MCF‐7 cells upon treatment with 3‐MA or stably infected with the PLAC8 lentiviral vector. Cell viability upon drug treatment was analysed by an MTS assay. G. Colony formation assay was performed to test MCF‐7 cell response to ADM upon treatment with 3‐MA or stably infected with the PLAC8 lentiviral vector. H. Drug sensitivity test for ADM in MCF‐7/ADMR cells upon treatment with RAPA or infected with the PLAC8 siRNA. I. Colony formation assay was performed to test MCF‐7/ADMR cell response to ADM upon treatment with RAPA or infected with the PLAC8 siRNA. J. The expression of PLAC8 and p62 in MCF‐7/ADMR cells infected with control siRNA or PLAC8 siRNA was analysed by Western blotting. K, L. The expression of Beclin‐1, LC3, PLAC8 and p62 in MCF‐7/ADMR cells infected with the PLAC8 lentiviral vector or co‐infected with p62 siRNA was analysed by Western blotting. M. Whole cell extracts from MCF‐7/ADMR were used for immunoprecipitation of the PLAC8 and p62. Input used as the loading control. The data are presented as the mean ± SD of three independent experiments. Student's *t* test was used for statistical analysis. *P < .05, **P < .01, ***P < .001

PLAC8 has been shown to promote autophagosome‐lysosome fusion by activating pro‐survival function of autophagy in pancreatic cancer. We then assessed PLAC8 regulation to autophagy in breast cancer cells. A significant elevation of p62, phosphor‐S6 and LC3‐II expression was observed in PLAC8 stable overexpression cells (C1 and C2 clones). Synchronously, after PLAC8 knockdown by siRNA in MCF‐7/ADMR cells, LC3‐II and p62 accumulation were reduced in the conditions of complete medium (Figure 5A). PLAC8‐induced decrease of autophagy was further confirmed, when compared with its parental cell line MCF‐7 (Figure 5B), and the inhibition of autophagy was released upon PLAC8 degradation in MCF‐7/ADMR (Figure 5C). Furthermore, we applied a mCherry‐GFP‐LC3 fusion construct tool for determining autophagy flux in MCF‐7 and MCF‐7/ADMR cells, increased content of red puncta is indicative of augmented autophagic flux (delivery of cargo into lysosomes), whereas yellow puncta (a merge of green and red channels) are indicative of autophagosome formation and accumulation. Overexpression of PLAC8 blocked autophagy activity, while PLAC8 silencing promoted autophagy flux via the autophagy reporter assay (Figure 5D,E).

Moreover, autophagy inhibitors 3‐MA promoted accumulation of autophagic vacuoles and enhanced the cytoprotective effect induced by PLAC8 overexpression to ADM cytotoxicity in the breast cancer cell (Figure 5F,G). In contrast, combined therapy of siRNA‐mediated PLAC8 depletion with RAPA treatment sensitized breast cancer cells to ADM treatment (Figure 5H,I). Next, we detected the expression levels of autophagy‐related molecules, to test whether PLAC8 affected ADM chemo‐sensitivity via specific autophagy‐related factors. Indeed, silencing the autophagy substrates of p62 significantly suppressed PLAC8 expression (Figure 5J‐L). Co‐immunoprecipitation (Co‐IP) experiments were performed to identify direct protein interactions with PLAC8, and p62‐PLAC8 was demonstrated as an interactive protein complex (Figure 5M). Here, our findings suggested that PLAC8 inhibited autophagy at least partially through the participation of p62 and then affected ADM resistance in breast cancer cells.

**FIGURE 6:**
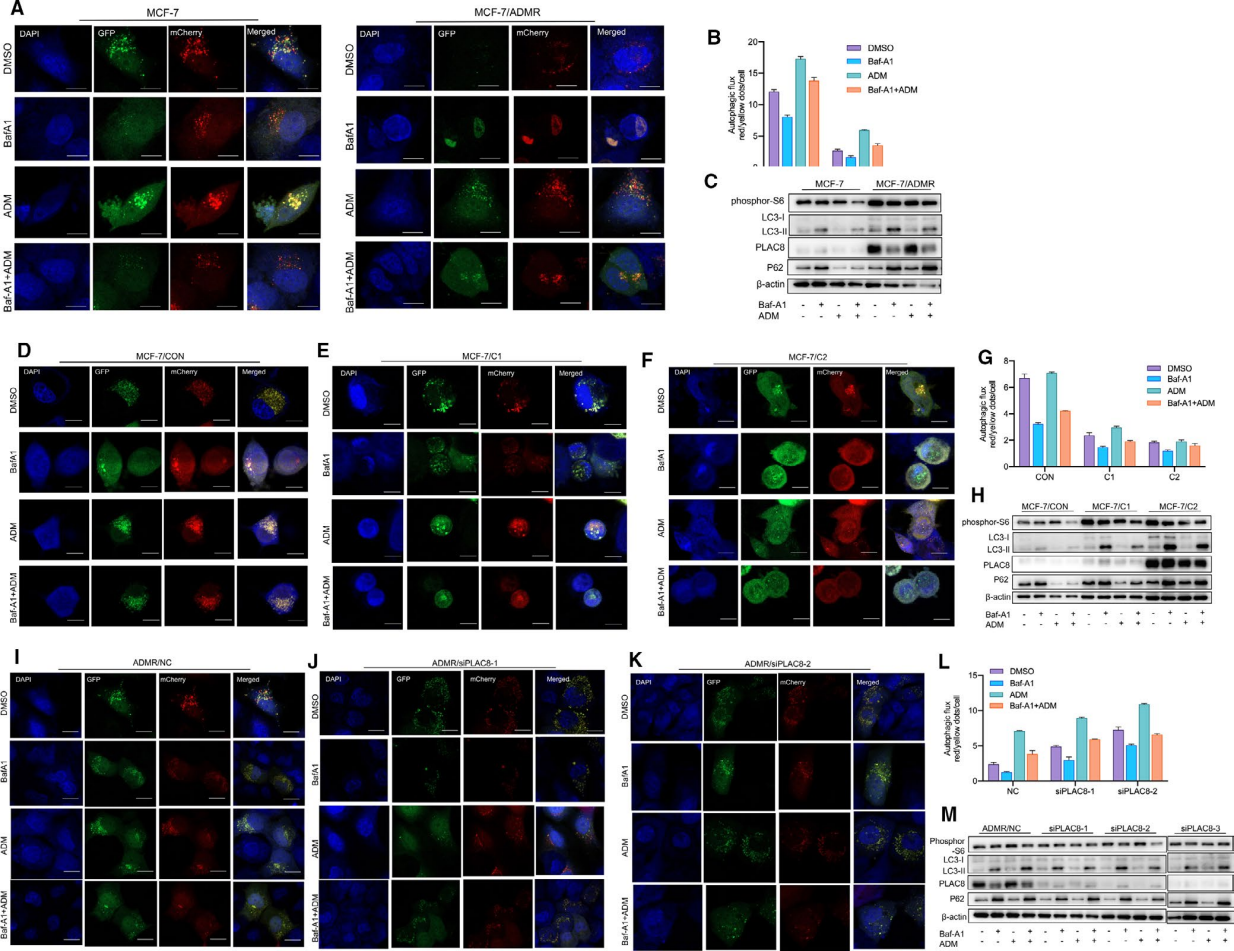
Autophagy activity after pharmacological inhibition of autophagy in breast cancer cells with PLAC8 overexpression or silencing A, B. MCF‐7 and MCF‐7/ADMR cells were treated with bafilomycin A1 (Baf‐A1) or DMSO, both in normal conditions and with ADR treatment, and autophagosome formation were measured by GFP‐LC3 puncta counting. Quantification of representative pictures showing the functionality of the sensor. Manual counting of red and yellow dots was performed. Autophagic flux is estimated as the ratio between red and yellow dots. C. The expression of phosphor‐S6, LC3, PLAC8 and p62 in MCF‐7 and MCF‐7/ADMR cells treated with Baf‐A1 or DMSO, both in normal conditions and with ADR treatment. GAPDH shown as a loading control. D‐G. MCF‐7/CON, MCF‐7/C1 and MCF‐7/C2 cells were treated with bafilomycin A1 (Baf‐A1) or DMSO, both in normal conditions and with ADR treatment, and autophagosome formation were measured by GFP‐LC3 puncta counting. Quantification of representative pictures showing the functionality of the sensor. Manual counting of red and yellow dots was performed. Autophagic flux is estimated as the ratio between red and yellow dots. H. The expression of phosphor‐S6, LC3, PLAC8 and p62 in MCF‐7/CON, MCF‐7/C1 and MCF‐7/C2 cells treated with Baf‐A1 or DMSO, both in normal conditions and with ADR treatment. GAPDH shown as a loading control. I‐L. ADMR/NC, ADMR/siPLAC8‐1 and ADMR/siPLAC8‐2 cells were treated with bafilomycin A1 (Baf‐A1) or DMSO, both in normal conditions and with ADR treatment, and autophagosome formation were measured by GFP‐LC3 puncta counting. Quantification of representative pictures showing the functionality of the sensor. Manual counting of red and yellow dots was performed. Autophagic flux is estimated as the ratio between red and yellow dots. M. The expression of phosphor‐S6, LC3, PLAC8 and p62 in ADMR/NC, ADMR/siPLAC8‐1, ADMR/siPLAC8‐2 and ADMR/siPLAC8‐3 cells treated with Baf‐A1 or DMSO, both in normal conditions and with ADR treatment. GAPDH shown as a loading control

We next measure autophagy flux after pharmacological inhibition of autophagy with bafilomycin A1(Baf‐A1) in normal conditions or co‐culturing with ADM. We have treated the MCF‐7 and MCF‐7/ADMR cells with bafilomycin A1 (Baf‐A1) or DMSO, both in normal conditions and with ADR treatment, and autophagosome formation was measured by GFP‐LC3 puncta counting and assessment of autophagy‐related proteins by Western blot analysis (Figure 6A‐C). Consistently with previously findings, autophagy flux was blocked in MCF‐7/ADMR, compared to its parental cell line. Increased LC3‐II and p62 accumulation were observed in the conditions of the complete medium in MCF‐7/ADMR cell. Baf‐A1, an autophagy inhibitor, suppressed autophagy in both MCF‐7 and MCF‐7/ADMR cell lines, and Western blot analysis revealed increased p62, phosphor‐S6 and LC3‐II protein accumulation in the Baf‐A1 group. Similarly, PLAC8 knocked‐down cells (ADMR/NC, ADMR/siPLAC8‐1 and ADMR/siPLAC8‐1) (Figure 6I‐M) as well as the C1 and C2 overexpressing clones cell lines (MCF‐7/CON, MCF‐7/C1 and MCF‐7/C2) (Figure 6D‐H) were treated with Baf‐A1 and/or ADM, and autophagosome formation was measured by GFP‐LC3 puncta counting and Western blot analysis. We observed suppressed autophagy flux and a significant increase of P62 and LC3‐II expression level in PLAC8 stable overexpression cells (MCF‐7/C1 and MCF‐7/C2 clones) (Figure 6D‐H). Synchronously, after PLAC8 knockdown by siRNA in MCF‐7/ADMR, LC3‐II and p62 accumulation were reduced, and PLAC8 silencing‐induced activation of autophagy was further confirmed (Figure 6I‐M). In addition, combined therapy of Baf‐A1treatment and PLAC8 overexpression synergistically suppressed autophagy process in MCF‐7 cell lines, Western blot analysis revealed increased p62 and LC3‐II protein accumulation in the Baf‐A1 and PLAC8 overexpression group.

### 3.6 PLAC8 silencing effectively abrogated breast cancer formation in vivo

**FIGURE 7:**
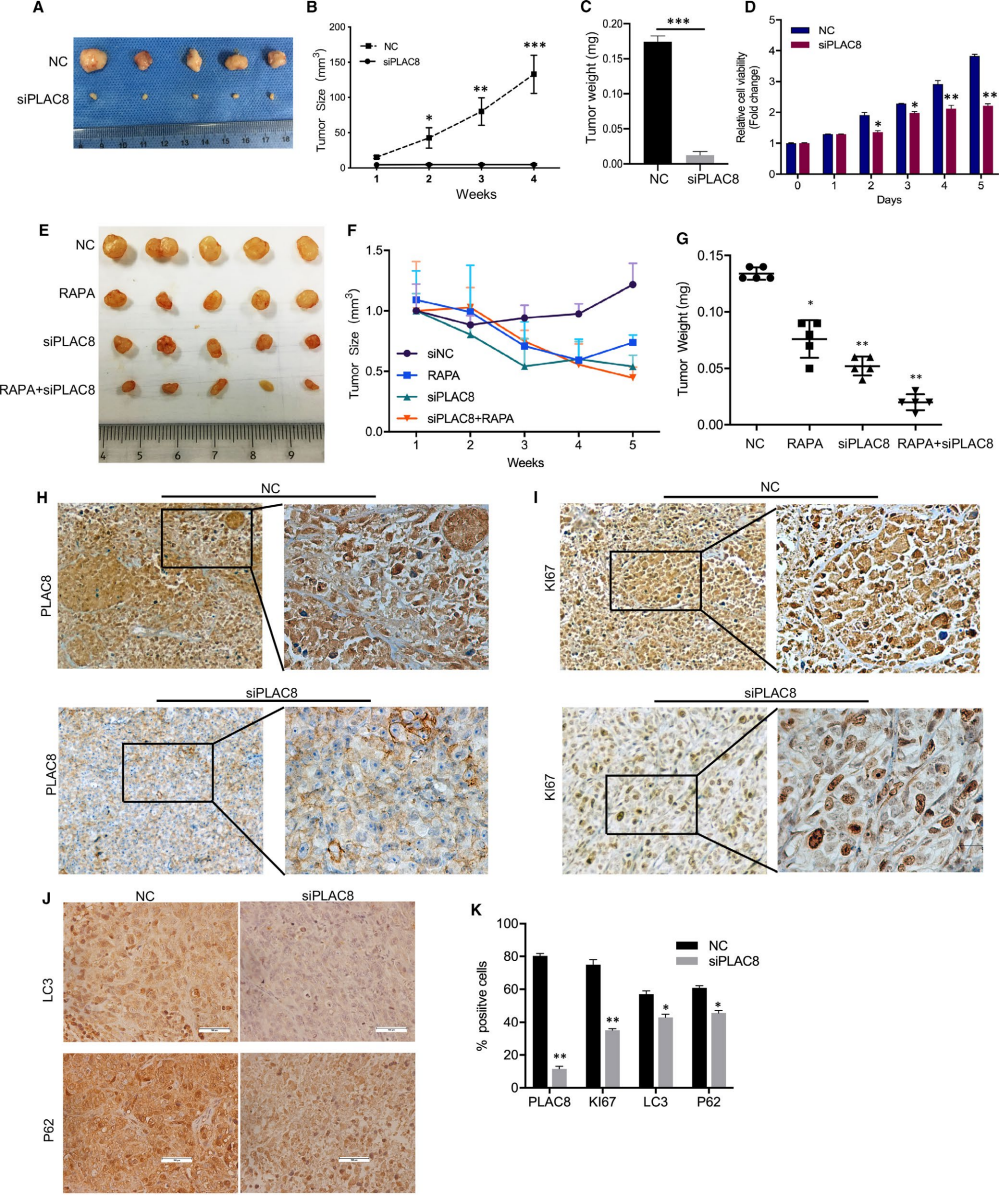
Effects of PLAC8 on breast tumour development in vivo A. PLAC8 silencing in MCF‐7/ADMR significantly inhibited xenografts growth. Shown are representative tumour images harvested at four weeks after implantation. B, C. Changes in tumour volume and tumour weight of MCF‐7/ADMR infected with the control siRNA or PLAC8 siRNA from week 1 to week 4. Data are shown mean ± SD. D. cell viability assay was performed and knockdown of PLAC8 inhibited cell proliferation of MCF‐7/ADMR without ADM treatment in vitro. E‐G. Combined therapy of rapamycin treatment and PLAC8 knockdown led to decreased xenografts tumours size and weight, and has a synergistic effect on inhibiting tumour growth. H, I. Immunohistochemistry analysis with PLAC8 antibody and Ki67 antibody on MCF‐7/ADMR cells infected with the control siRNA or PLAC8 siRNA. Representative sections are shown. Black bars = 50 μm. J. Immunohistochemistry analysis of LC3 and p62 expression in MCF/ADR (CON) and MCF‐7/ADMR (siPLAC8) groups. K. Per cent of PLAC8+, Ki67+, LC3+ and p62+ cells were quantified. Each bar represents the mean ± SD of three independent experiments. *P < .05, **P < .01, ***P < .001

PLAC8 knockdown in animal models abrogated breast tumour growth supporting an oncogenic role. In animal models, MCF‐7/ADMR cells with PLAC8 silencing grew significantly slower and smaller than their control (Figure 7A,7B). Significant differences were found in tumour volume between the PLAC8 silencing groups compared with those in the vector groups four weeks post‐implantation (Figure 7C). The cell viability assay was performed, and we presented that knockdown of PLAC8 inhibited cell proliferation of MCF‐7/ADMR without ADM treatment in vitro (Figure 7D). In addition, we applied one autophagy modulators rapamycin to further analysis the effect of autophagy on xenograft growth. Consistently with previously in vitro experiments, combined therapy of rapamycin treatment and PLAC8 knockdown led to decreased xenografts tumours size and weight, and has a synergistic effect on inhibiting xenograft growth (Figure 7E‐G).

As expected, the PLAC8 expression was relatively decreased in the PLAC8 knockdown groups (Figure 7H). Results from the IHC staining of Ki67 showed decreased tumour proliferation ability in the PLAC8 knockdown groups (Figure 7I). Furthermore, PLAC8 knockout lead to decreased expression of LC3 and P62 protein (Figure 7J,7K). Here, animal models further supported that PLAC8 knockdown in MCF‐7/ADMR cells suppressed tumour formation ability, and the process of autophagy was promoted in PLAC8 silencing groups.

## 4 DISCUSSION

ADM remains to be one of the most widely used chemotherapy agents in the treatment of early and advanced breast cancer. However, drug resistance has limited the effectiveness of the agent in cancer treatment. Our study highlighted that breast cancer cells with high PLAC8 expression responded weakly to ADM treatment, and PLAC8 was necessary and sufficient in inducing ADM‐resistant phenotype. PLAC8 may alter the ADM sensitivity of breast cancer cells by modulating autophagy process. And combined treatment of autophagy inducer RAPA and PLAC8 knockdown could efficiently reverse ADM resistance in breast cancer.

The lysosomal protein PLAC8 localizes to the inner surface of the plasma membrane and interacts with other cellular components to modulate autophagy, cell cycle arrest and apoptosis. The function of PLAC8 differs based on the cell type. For example, ectopic expression PLAC8 protects fibroblasts but induces apoptosis in epithelial cells.^17^, ^18^ In cancer cells, PLAC8 induced epithelial‐mesenchymal transition, and cell cycle was reported to be regulated by PLAC8 in pancreatic cancer cells.^19^, ^20^ PLAC8 also plays a role in autophagosome‐lysosome fusion by initiating oncogenic autophagy in pancreatic cancer.^13^ In our studies, high PLAC8 expression is correlated with breast cancer resistant to ADM and poor outcome. Moreover, silencing PLAC8 expression enhances ADM response in MCF‐7/ADMR cells, whereas PLAC8 overexpression causes ADM resistance in MCF‐7 cells. Our results further added evidence to the versatility of PLAC8 in cellular functions. PLAC8 may be used as a novel biomarker in the prediction of patient responsiveness to ADM and serves as a unique therapeutic target for overcoming ADM resistance in breast cancer.

Autophagy is an evolutionarily conserved catabolic process, in which cellular proteins and organelles are engulfed by autophagosomes, digested in lysosomes and recycled to sustain cellular metabolism.^21^ Autophagy has dual roles in cancer, acting as a tumour suppressor by preventing the accumulation of damaged proteins and organelles and a mechanism of cell survival to promote the growth of established tumours.^22^, ^23^ Autophagy process is also a promising target for drug development in cancer. Autophagy can be monitored using markers proteins, namely LC3 and p62. During autophagosome formation, cytosolic LC3 I is lipidated, recruited to the membranes and designated as LC3‐II. Lipidated LC3‐II migrates faster than LC3I on polyacrylamide gels, and the ratio of autophagosomal LC3‐II to cytosolic LC3I is used as an indicator of autophagy.^24^, ^25^ p62 is also a cargo recognition protein that binds to misfolded aggregate proteins in autophagosomes and is degraded in autolysosomes.^26^ Previously, PLAC8 is found to be correlated with autophagosome and promotes autophagy by facilitating autophagosome‐lysosome fusion. Our experiments suggested that PLAC8 may affect ADM sensitivity by modulating autophagy in breast cancer.

Specifically, autophagy activators and inhibitors are an active area of investigation in cancer therapeutics, and some inhibitors are being examined for their efficacy in clinical trials. An increased understanding of autophagy in cancer is important for its optimal utilization to enhance the effects of chemotherapy and improve clinical outcomes of treatment in cancer patients. Overall, these data demonstrate that PLAC8 overexpression can collaborate with p62 to suppress autophagy. The molecular basis of autophagy dependence in cancer remains unknown, and identifying genes critical for this process in cancer cells is crucial for exploiting the inhibition of autophagy as an anticancer strategy. These findings suggest that PLAC8 and/or autophagy inducers combined with chemotherapy may be a new strategy for the treatment of ADM resistance in patients with breast cancer.

## 5 CONCLUSION

PLAC8 plays critical role in the development and progression of breast cancer. In this study, we examined the clinical significance and biological effects of PLAC8 in breast cancer progression and ADM sensitivity regulation. Our findings proposed that PLAC8 contributed to ADM resistance in breast cancer via modulation of autophagy. Thus, understanding the novel function of autophagy may allow us to develop a promising therapeutic strategy to enhance the effects of chemotherapy and improve clinical outcomes in the treatment of cancer patients.

## CONFLICT OF INTEREST

The authors declared that they have no conflicts of interest to this work.

## AUTHOR CONTRIBUTION

**yongxia chen:** Conceptualization (equal); Data curation (equal); Methodology (supporting); Project administration (equal); Writing‐original draft (equal); Writing‐review & editing (equal). **Yunlu Jia:** Formal analysis (equal). **misha mao:** Methodology (equal). **Yifeng Gu:** Data curation (supporting); Formal analysis (supporting). **chenpu xu:** Resources (supporting). **jingjing yang:** Formal analysis (supporting). **wenxian Hu:** Resources (supporting). **Jun Shen:** Software (supporting). **dengdi Hu:** Resources (supporting). **cong chen:** Resources (supporting). **zhaoqing li:** Resources (supporting). **lini chen:** Resources (supporting). **Jian Ruan:** Visualization (supporting). **Peng Shen:** Supervision (supporting). **Jichun Zhou:** Resources (supporting). **qun wei:** Data curation (supporting); Methodology (supporting); Project administration (supporting). **linbo wang:** Funding acquisition (supporting); Project administration (lead).

## ETHICS STATEMENT

For the analysed tissue specimens, all patients provided informed consent to use excess pathological specimen for research purposes. The protocols employed in this study and the use of human tissue were approved by the Ethics Committee of the Sir Run Run Shaw Hospital, affiliated with Zhejiang University and conducted in full accordance with the ethical principles cited in the World Medical Association Declaration of Helsinki and local legislation.
